# Kawasaki Disease or Cat Scratch Disease? A Diagnostic Dilemma: A Paediatric Case Report and Literature Review

**DOI:** 10.31138/mjr.100624.ahe

**Published:** 2025-06-30

**Authors:** Daiva Gorczyca, Daniel Lewandowski, Jacek Postępski

**Affiliations:** 1 Centre for Chronically Sick Children Berlin, Charité – Universitätsmedizin Berlin, Berlin, Germany,; 2 Department of Trauma & Orthopaedics, University Hospital of Wales, Cardiff, United Kingdom,; 3 Department of Paediatric Pulmonology and Rheumatology, Medical University of Lublin, Lublin, Poland

**Keywords:** Bartonella henselae, cat-scratch disease, children, intravenous immunoglobulin, Kawasaki disease, spleen

## Abstract

**Objective:**

Kawasaki disease (KD) and atypical cat-scratch disease (CSD) can manifest with fever and similar non-specific symptoms.

**Methods:**

We report the case of an immunocompetent child who presented with signs and symptoms consistent with KD, which overlapped with those of atypical CSD (hepatosplenic form). Subsequently, we conducted a literature review to identify paediatric cases of KD and CSD.

**Results:**

We present the case of a 6-year-old girl with fever, abdominal pain, non-purulent bilateral conjunctivitis, maculopapular rash on the trunk, bilateral cervical lymphadenopathy, and oral mucosa erythema, meeting KD diagnostic criteria**.** Echocardiography revealed coronary artery dilatations. Despite initial intravenous immunoglobulin (IVIG) treatment eighteen hours later the general condition worsened, with the recurrence of fever, diffuse myalgia, severe abdominal pain, and vomiting. A detailed history revealed a cat scratch three weeks before onset, along with an erythematous nodule on the thumb, axillary lymphadenopathy, a typical hypoechoic splenic lesion in abdominal ultrasonography, and highly elevated IgM and IgG antibodies for *Bartonella henselae* titres, leading to a diagnosis of atypical CSD. Successful treatment involved a three-month course with erythromycin. Our literature review revealed five cases of co-occurring KD and CSD and six cases where CSD mimicked autoimmune diseases or malignancies.

**Conclusion:**

The presented case illustrated the expanding spectrum of *B. henselae* infection and emphasised the importance of including it in the differential diagnosis of KD and prolonged fever syndromes. We suggest incorporating abdominal ultrasonography into the initial diagnostic workup, considering it to be essential before empiric therapy initiation.

## INTRODUCTION

Kawasaki disease (KD) is a vasculitis of medium-sized vessels, primarily affecting coronary arteries, potentially leading to their dilatation. It typically occurs in children under 5 years old, manifesting acute febrile disease with polymorphic rash, conjunctival injection, oropharyngeal and lip mucositis, tongue papillitis, erythema and edema of the hands and feet, as well as unilateral cervical lymphadenopathy.^1^ In the absence of an available and affordable diagnostic test, a detailed clinical history and physical examination remain crucial for diagnosing KD. It is important to recognise that delayed diagnosis or misdiagnosis of KD can result in an increased risk of cardiac complications, e.g., coronary artery aneurysms, which develop in approximately 25% of untreated cases of KD.^1^

The bacterium *Bartonella henselae*, an aerobic, intra-cellular, gram-negative bacillus, causes cat-scratch disease (CSD) that commonly affects children and teenagers. In typical CSD, the incubation period ranges from 3 to 10 days, and a papule appears at the site of inoculation approximately 4 to 6 days after the animal scratch.^2^ The regional lymphadenopathy usually develops 1 - 3 weeks after the initial skin findings. This is often associated with constitutional symptoms such as fever, malaise, and night sweats. The disease usually resolves spontaneously within a few weeks.

An extra-nodal manifestation is collectively known as atypical or systemic CSD. It commonly presents with fever of unknown origin, hepatosplenic disease, neurological, cardiac, Parinaud’s syndrome, or musculoskeletal symptoms.^3^ It remains unclear why some patients suffer from atypical manifestations involving visceral organs.

We report a case of an immunocompetent child who presented signs and symptoms consistent with KD, which overlapped with those of atypical CSD (hepatosplenic form). Subsequently, we searched for pediatric cases of KD and CSD published in the literature.

In our case, signed informed consent was obtained from the parents of the patient. The local ethics committee approved the report protocol.

## CASE DESCRIPTION

**Figure 1.**
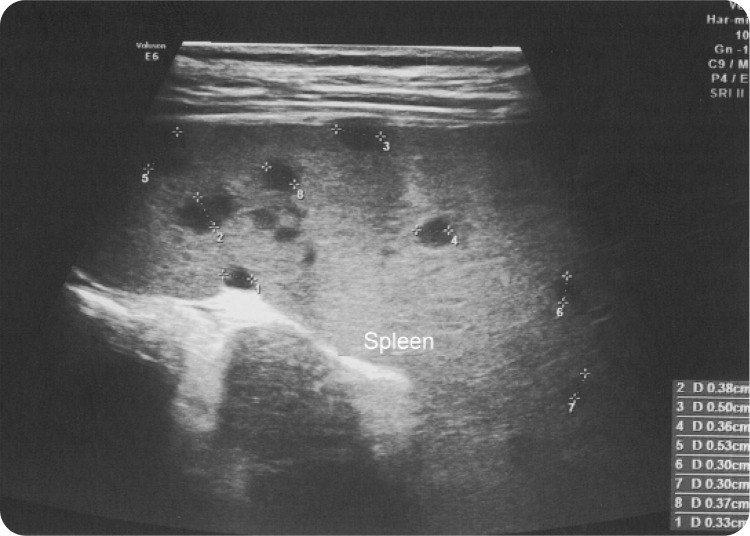
Spleen ultrasound with multiple hypoechoic areas at diagnosis of CSD.

A 6-year-old Caucasian girl was admitted to the hospital due to a fever (39.2^o^C) lasting 2 days, associated with abdominal pain, non-purulent bilateral conjunctivitis, maculopapular rash on the trunk, bilateral cervical lymphadenopathy, and erythema of the oral mucosa. Initial laboratory tests were within normal ranges, except for elevated C-reactive protein levels (71.4 mg/L; normal range <6 mg/L) and erythrocyte sedimentation rate (54 mm/h; normal range 0–20 mm/h). Serologic testing was negative for Epstein-Barr virus, cytomegalovirus*, Mycoplasma pneumoniae*, and *Chlamydia pneumoniae*. Cultures of blood samples and urine were negative. Testing for anti-nuclear antibodies (ANA), anti-(double stranded)-DNA antibodies (anti-ds-DNA), extractable nuclear antigen antibodies (ENA), anti-proteinase 3 (anti-PR3), and anti-myeloperoxidase (anti-MPO) antibodies were negative. Despite antibiotic therapy with intravenous amoxicillin, the fever persisted. Due to this reason, an expanded differential diagnosis was performed, including infectious and non-infectious conditions. Echocardiography revealed dilated left and right coronary arteries, of 3 mm and 6 mm, respectively. On the eighth day of fever, KD was diagnosed. On the same day blood test showed an anemia for age (haemoglobin 8.6 g/dl; normal range: 11.9–15.0), moderately raised ALT level (68 IU/ml; normal range 0–48), leukocyturia (21–30 WBC/hpf). The patient received intravenous immunoglobulins (IVIG) at a dose of 2 g/kg together with aspirin at a dose of 80 mg/kg/day divided into four doses. A short-term improvement in the general condition and a transient normalisation of the body temperature were observed. Eighteen hours after IVIG infusion, the general condition of the patient worsened, with the reoccurrence of fever, diffuse myalgia, severe abdominal pain, and vomiting. Abdomen ultrasonography revealed an enlarged liver, hypoechoic areas in the spleen (**Figure 1**), and enlarged left axial lymph nodes, with the largest node measuring 2.5x1.5 cm. An extended history showed that the patient had been scratched on the left thumb by a cat three weeks before admission. Upon examination, an erythematous nodule (2 mm) on the thumb was noted. Serological analyses by the indirect fluorescent antibody (IFA) method detected the positive presence of both IgM 1:160 (normal range <1:20) and IgG 1:1024 (normal range <1:64) antibodies to *B. henselae*. Treatment with erythromycin (40 mg/kg/day administered orally in four divided doses) for 3 months was introduced. Two weeks later, abdominal ultrasonography displayed a normal liver and a regression of spleen lesions. A 6-month follow-up showed no evidence of local or systemic relapse of *B. henselae* infection, and no coronary artery abnormalities were observed.

### Discussion of similar published cases

We performed a search using PubMed and Google Scholar. Initially, we focused on identifying cases resembling KD and *B. henselae* infections using keywords such as “Kawasaki disease”, “Cat Scratch Disease”, “Bartonella henselae”, “spleen”, “liver”, “disseminate”, “systemic”, and “mimicker” with detailed control of references. There were no language restrictions. Additionally, we expanded our literature search to determine whether an atypical (sometimes referred to as extra-nodular, systemic, or disseminated) *B. henselae* infection could present as a mimic of other autoimmune diseases in pediatric patients. Keywords for this extended search included “Cat Scratch Disease”, “Bartonella henselae”, “spleen”, “liver”, “disseminate”, “systemic”, “child”, and “mimicker”. Demographic data (sex, age), epidemiological data, clinical presentations, laboratory findings, radiologic images, clinical course, and treatments were reported and discussed.

It is important to note that Tsuchida et al. published an abstract in Japanese from a conference (319th Kanagawa Regional Meeting of the Japanese Pediatric Society) rather than a full paper.^4^ Despite the limited accessibility of the abstract in English and full text, we included the available data from this abstract. However, we excluded publications referenced by Ma M et al. in the 11^th^ and 12th positions due to the unavailability of the abstracts and/or full texts.^5^

Our case of typical KD, which overlapped with the hepatosplenic form CSD, and the cases from the literature review, highlight the diagnostic complexities in both KD and CSD because these diseases often present with nonspecific symptoms. Through our literature search, we identified five cases describing the co-occurrence of KD and CSD (**Table 1**).

**Table 1.** Reported cases of Kawasaki disease with Cat Scratch Disease (our case included).

	**Index Case**	**Case 2 (5)**	**Case 3 (6)**	**Case 4 (4)**	**Case 5 (18)**	**Case 6 (18)**
**Age (years)/Sex**	6/F	5/F	5/F	7/F	4/M	8/M
**Contact to cat**	yes	yes	yes	no, with a hamster	yes	-
**History of cat scratch**	yes	yes	yes	no	yes	-
**Time from cat scratch to first symptoms**	3 weeks	10 days	several months	-	-	-
**Fever, duration (days)**	8 days	5 days	11 days	3 days	7 days	yes
**Bilateral conjunctivitis, bulbar, without exudate**	yes	yes	-	yes	yes	yes
**Changes of lips or oral mucosa**	erythema of oral mucosa	sore throat, erythema of the lips	-	yes	no	-
**Rash**	maculopapular rash on the trunk	pale erythema on the anterior surface of the trunk	-	yes	generalised maculopapular rash	yes
**Lymphadenopathy**	bilateral cervical, left axilla	no	left axilla	cervical	unilateral cervical	-
**Changes to extremities**	-	erythema and oedema of the palms and soles	-	desquamation on the 21st day	-	yes
**Coronary artery lesions**	left and right CAL	no	left CAL, repeated echo normal	-	no	-
**Abdominal pain**	crampy abdominal pain	-	crampy abdominal pain, nausea	-	-	
**Eye manifestations**	-	-	-	-	bilateral anterior uveitis	-
**Musculoskeletal’**	diffuse myalgia		myalgia, chest pain			arthralgia
**Hepatomegaly**	yes	-	-	-	-	-
**WBC count (×109/L)**	8.2	15.9	10.9	-	-	-
**Initial CRP (mg/L)**	71	75	74	-	124	-
**ESR (mm/h)**	54	-	-	-	120	-
**Bartonella henselae Antigen Titre IFA (IgM/IgG)**	1:160/1:1024	1:40 / 1:512	-	-	IgG 1:100	IgG 1:100
**Microbiology (culture, PCR)**	-	-	PCR positive	-	-	-
**Abdominal USG**	multiple granulomatous lesions in the spleen, hepatomegaly	multiple lesions suggesting micro-abscesses in the spleen	first abdominal USG as normal; 2 weeks later numerous small, round, hypoechoic lesions in the liver	-	mild splenomegaly	-
**Author’s Interpretation**	co-morbidity: complete KD and atypical CSD	atypical CSD mimicking complete KD	atypical CSD mimicking incomplete KD	CSD mimicking complete KD	incomplete KD, CSD mistakenly diagnosed due to false-positive serology following IVIG therapy	incomplete KD, CSD mistakenly diagnosed due to false-positive serology following IVIG therapy
**Treatment of KD**	IVIG 2 g/kg and aspirin	IVIG 2 g/kg and aspirin, but the fever persisted	IVIG two times, because the fever persisted, and aspirin	IVIG two times, aspirin	IVIG 2 g/kg to reduce the risk of KD consequence	IVIG
**Treatment of CSD**	amoxicillin, erythromycin 3 months	ceftriaxone-azithromycin 10 mg/kg, as second-line trimethoprimsulfamethoxazole	no data	azithromycin - ampicillin/sulbactam - meropenem - mitomycin	doxycycline 7 days	no data
**Clinical resolution/time to cure**	Abdominal USG displayed a normal liver two weeks later and a regression of spleen lesions. A 6-month follow-up showed no local or systemic relapse of Bartonella infection, and no coronary artery abnormalities were observed.	The fever resolved on the 8th day of hospitalisation. 10 days after the complete resolution of the granulomatous lesion in the spleen	no data	no data	recovery	recovery

CRP: C-Reactive Protein; CSD: cat scratch disease; ESR: Erythrocyte Sedimentation Rate; IFA: immunofluorescence assays; IVIG: intravenous immunoglobulins; KD: Kawasaki disease; PCR: polymerase chain reaction; USG: ultrasonography; WBC: White Blood Cell.

In our case, the initial clinical presentation, characterised by the presence of four of the five main clinical symptoms, combined with the detection of the dilatation of coronary arteries, supported the diagnosis of complete KD. The diagnosis of atypical (systemic) CSD was based on the history of a cat scratch three weeks before fever onset, along with the identification of an erythematous nodule on the thumb, prolonged fever, axillary lymphadenopathy, a typical splenic lesion in abdominal ultrasonography, and positive *B. henselae* IgM, IgG serology. We have identified two similar cases of 5-year-old previously healthy girls initially diagnosed with KD and treated with IVIG along with aspirin.^5,6^ Due to persistent fever after the second dose of IVIG, a repeat abdominal ultrasonogram examination was performed and demonstrated numerous small, round, hypoechoic lesions in the spleen^5^ and in the liver.6 Although we lacked the abdominal ultrasonogram before the KD diagnosis and treatment with IVIG, in these cases, the initial abdominal ultrasonogram was normal. Fusani et al., in a retrospective study from a Children’s Hospital in Italy, showed that the systemic CSD form manifested with the classic red-brown non-tender papule (33.3%), more than 14 days of fever (median 17 days), abdominal pain (22.2%), rash (44.4%), asthenia (66.7%), and weight loss (44.4%).^7^ Notably, in this cohort, in all patients with systemic CSD, an abdominal ultrasound revealed alterations compatible with CSD. In our identified case series (**Table 1**), fever was the most frequent clinical manifestation, observed in all patients, with or without rash in 5 out of 6 cases, lymphadenopathy in 4 out of 6 cases, and crampy abdominal pain and myalgia were each reported in 2 out of 6 cases. However, patients with fever, rash, and lymphadenopathy can be misdiagnosed as having KD instead of infectious (Mycoplasma-induced rash and mucositis, Staphylococcal scalded skin syndrome, BCGosis) or non-infectious (systemic onset juvenile idiopathic arthritis, multisystemic inflammatory syndrome in children (MIS-C) associated with SARS-CoV2) diseases.^8^ In 3 out of 6 cases, abdominal ultrasonography was very useful in diagnosing the hepatosplenic form of CSD, as it can identify typical hypoechoic lesions in the spleen and/or liver.^3,9^ Deregibus et al., in a study conducted at a tertiary care hospital in Argentina, found pathological abdominal ultrasonography in 85.7% of CSD cases.^10^

Our patient, as well as patients from our literature review, have an elevated C-reactive protein level (CRP) (≥ 30 mg/L) and erythrocyte sedimentation rate (ESR) (≥ 40 mm/hour). A Scientific Statement for Health Professionals from the American Heart Association included CRP ≥ 30 mg/L and ESR ≥ 40 mm/hour among supportive laboratory tests for diagnosing incomplete KD.^1^ Fusani et al. reported increased CRP (median 23.4 mg/L, IQR=7.3–44.7) and ESR (median 31 mm/hour, IR=26.25–66.5 mm/hour) levels in the systemic CSD group.^7^

Atypical (systemic) CSD can be easily mistaken for other diseases given its dissemination through the bloodstream and diverse manifestations of symptoms. We identified six cases reported in the literature where CSD in children mimicked autoimmune diseases or oncological processes (**Table 2**). Atypical CSD has been interpreted as a mimicker of systemic lupus erythematosus,^11^ Langerhans cell histiocytosis,^9^ sarcoidosis,^12^ or spondylitis.^13^ In another case, *B. henselae* has been implicated as a trigger for systemic juvenile idiopathic arthritis,^14^ or autoimmune thrombocytopenia, which was noted as a complication of CSD.^15^

**Table 2.** Cases collected from a literature review on cat scratch disease in children, as an imitator of another disease.

	**Case 1 (11)**	**Case 2 (9)**	**Case 3 (12)**	**Case 4 (14)**	**Case 5 (15)**	**Case 6 (13)**
**Age (years)/Sex**	11/F	11/F	7/F	4/F	9/M	5/M
**Contact to cat**	yes	yes	yes	yes	-	yes
**History of cat scratch**	-	yes	no	yes	-	unknown
**Time from cat scratch to first symptoms**	-	-	unknown	1 month	-	2 months
**Fever**	-	-	3 weeks	3 weeks	Eleven days before fever (39°C) for three days, with a successive spontaneous regression	4 weeks
**Fatigue**	-	yes	yes	-	-	-
**Weight-loss**	-	-	yes	-	-	-
**Night sweats**	-	-	yes	-	-	-
**Headache**	-	yes		-	-	-
**Lymphadenopathy**	-	-	hilar lymph nodes	-	cervical	in the upper right leg
**Abdominal pain**	-	-	-	-	-	yes
**Eye manifestations**	neuroretinitis	-	unilateral conjunctivitis 3 days, unilateral anterior granulomatous uveitis	-	-	-
**Musculoskeletal**	-	8 cm large soft tissue mass on the left temporal bone	arthralgias	myalgia of the legs	-	low back pain without neuro damage
**Rash**	-	-	erythematous popular rash	no	purpura on limbs and trunk	-
**WBC count ((×109/L)**	-	-	18	6.9	8.9	14.6
**Initial CRP (mg/l)**	-	68	80	30–90	normal range	49
**ESR (mm/h)**	-	95	86	100–150	normal range	-
**Immunodeficiencies**						
**screening**	-	-	negative	negative	-	negative
**Autoimmune diagnostic**	-	--	negative	negative	negative	negative
**B. henselae Antigen Titre**			1:32 / 1:256			negative/1:256
**IFA (IgM/IgG)**	-	1:200 / 1:6400		negative/1:4096	IgG 1:2048	
**Microbiology (culture, PCR)**	-	-	PCR negative	PCR negative	-	blood PCR negative, lymph node PCR positive
**Abdomen USG**	-	several hypodense lesions in the liver and spleen	small hypoechoic lesions in the liver and spleen; subsequent MRI revealed multiple focal lesions in the organs above in keeping with granulomata; echocardiogram normal	low echoic lesions in the liver and spleen on day 38th	-	hepatic and splenic hypoechogenic lesions without hepatosplenomegaly; nine months later no lesions
**Autoimmune disease with the most relevant results**	Systemic lupus erythematosus positive ANA, hematuria	Langerhans cell histiocytosis a circumscribed lytic skull lesion, histopathology revealed necrosing inflammation without evidence for LCH	Sarcoidosis serum Ca 11.8 mg/dl, ACE 120 mg/dl	Systemic Juvenile Idiopathic Arthritis hypergammaglobulinemia, high complement titres, and an elevated titre of antinuclear antibody 1:80	Autoimmune thrombocytopenia platelet count 7 × 109/L, coagulation parameters in the reference range	Spondylitis of the D6 vertebra in MRI
**Author’s Interpretation**	-	Mimicker	Mimicker	B. henselae as trigger of autoimmune disease	As a complication of CSD	Mimicker
**Treatment of CSD**	-	Azithromycin 12 weeks	intravenous gentamicin for 7 days, followed by oral ciprofloxacin for 21 days. A combination of topical steroids and tobramycin for a total period of 6 weeks was used to control uveitis	-	Azithromycin 14 days	intravenous rifampicin for 6 weeks and azithromycin for 4 weeks
**Treatment of mimicker**	Methylprednisolone	none	none	Aspirin 41 days	IVIG 1/kg/die for two days	none
**Clinical resolution/time to cure**	-	Fever episodes, malaise, and headaches completely resolved within 3 months, first signs of regression of hepatic lesion after 6 months, 12 months later complete	Fever and constitutional symptoms improved within 72 hours of commencing therapy	11 months later no changes in sonography	end of antibiotic therapy a total regression of cervical lymphadenopathy. After 14 days from hospitalisation, the platelet count was 211x109/L and the patient was asymptomatic	The fever decreased two days after the correct antibiotic treatment, and a love grade fever continued for three weeks. Nine months later no changes in USG. Fatigue for two months

ANA: antinuclear antibodies; CRP: C-Reactive Protein; CSD: cat scratch disease; ESR: Erythrocyte Sedimentation Rate; IFA: immunofluorescence assays; IVIG: intravenous immunoglobulins; KD: Kawasaki disease; MRI: magnetic resonance imaging; PCR: polymerase chain reaction; USG: ultrasonography; WBC: White Blood Cell.

We believe that both our diagnoses, KD and CSD, were correct and we were dealing with a coincidence in timing when the child developed KD and, shortly thereafter, symptoms of atypical (systemic) CSD. To the best of our knowledge, the time for the development of granulomatous lesions in the liver and spleen in the case of hepatosplenic forms of CSD in immunocompetent patients has not been described in the literature. Johnson et al. reported that the IgM titre against *B. henselae* became positive by day eleven of the illness defined by the onset of fever and negative by day 40.^16^

IVIG in conjunction with aspirin, is the treatment of choice for Kawasaki disease.^1^ The main mechanisms of the beneficial effects of high dose (> 1 g/kg body weight) IVIG in autoimmune disorders include neutralisation of the functional activity of various autoantibodies and/or inhibition of binding of the autoantibodies to their respective autoantigens, inference with activation of complement and the cytokine network, regulation of T and B cell growth, differentiation, as well as effector functions that provide immunodepression.^17^ IVIG therapy could therefore be associated with increased susceptibility to infections and latent infection reactivation, but currently, a lack of literature data fails to confirm this hypothesis. Yakubowski et al. reported two cases of atypical KD with only positive IgG titres for CSD and suggested that the administration of IVIG provides for the misdiagnosis of CSD.^18^ However, the authors have not provided enough epidemiological data and the timing of serology tests was unspecified in both cases. There was also no information regarding fever recurrence after the initial doses of IVIG**.** In our case and others from our literature review,^5,6^ *B. henselae* serology was performed after the first IVIG infusion in children who did not respond to IVIG administration and were positive for specific IgM and IgG antibodies titres. The systemic literature review by Lopez et al. has reported cases of *Bartonella henselae* infection in pediatric solid organ recipients treated with mycophenolate mofetil, tacrolimus, and prednisolone.^19^ Clinically, patients have more often presented with typical CSD, and in the case of atypical CSD hepatosplenic granulomatous lesions, they occur in immunocompetent patients. The hepatic peliosis seen in immunosuppressed patients, especially in HIV patients, was not reported. Incidences of CSD with regional lymphadenopathy and extra-nodular CSD in adults with rheumatologic diseases during therapy with biologic disease-modifying antirheumatic drugs (bDMARDs) were reported previously.^20–24^ To the best of our knowledge, there are no reports in the pediatric population.

The therapeutic approach for *B. henselae* infection is not standardised and for atypical (systemic) *Bartonella* infections in children includes macrolides (e.g., erythromycin, azithromycin), rifampicin, or doxycycline for children older than 8 years of age.^25,26^ The optimum duration of antibiotic therapy has not been determined. Our patient tolerated 3 months of therapy with erythromycin well, and a complete recovery was made.

In conclusion, the presented case of a child with KD overlapping with the hepatosplenic form of CSD illustrated the expanding spectrum of *B. henselae* infection and emphasised the importance of considering this disease in the differential diagnosis of KD and prolonged fever syndromes. We propose the incorporation of abdominal ultrasonography into the initial diagnostic workup considering it to be essential before empiric therapy initiation.

## DECLARATION OF INTERESTS

The authors declared no funding and conflicts of interest.

## AUTHOR CONTRIBUTIONS STATEMENT

DG conceived of and designed the study. DL prepared the case report. JP was involved in planning and supervising the manuscript. All authors contributed to the interpretation of results, participated in writing the manuscript, critically reviewed the manuscript, and approved the final version for submission.

## DISCLAIMER

I, the Corresponding Author, declare that his manuscript is original and has not been copied, submitted, or published elsewhere.
